# Epitope Analysis of an Anti-Whale Podoplanin Monoclonal Antibody, PMab-237, Using Flow Cytometry

**DOI:** 10.1089/mab.2019.0045

**Published:** 2020-02-14

**Authors:** Yusuke Sayama, Masato Sano, Mika K. Kaneko, Yukinari Kato

**Affiliations:** ^1^ Department of Antibody Drug Development, Tohoku University Graduate School of Medicine, Sendai, Japan.; ^2^ New Industry Creation Hatchery Center, Tohoku University, Sendai, Japan.

**Keywords:** whale, podoplanin, monoclonal antibody, epitope, PMab-237

## Abstract

Podoplanin (PDPN) is a small mucin-type transmembrane glycoprotein, which was first discovered in podocytes of the kidney. PDPN is a specific lymphatic endothelial marker and is also known as T1alpha, a marker of lung type I alveolar cells, or Aggrus, a platelet aggregation-inducing factor. PDPN possesses three platelet aggregation-stimulating (PLAG) domains and PLAG-like domains (PLDs), which bind to C-type lectin-like receptor-2. Previously, we developed a novel anti-whale PDPN (wPDPN) monoclonal antibody (mAb) PMab-237 using the Cell-Based Immunization and Screening (CBIS) method and the RIEDL tag of Arg-Ile-Glu-Asp-Leu sequence. PMab-237 detected wPDPN by flow cytometry, western blot, and immunohistochemical analyses. However, the specific binding epitope of PMab-237 for wPDPN remains unknown. In this study, deletion mutants and point mutants of wPDPN with N-terminal RIEDL tag were produced to analyze the PMab-237 epitope using flow cytometry. The analysis of deletion mutants showed that the N-terminus of the PMab-237 epitope exists between the 80th amino acid (AA) and the 85th AA of wPDPN. In addition, the analysis of point mutants demonstrated that the critical epitope of PMab-237 includes Leu82 and Thr84 of wPDPN, indicating that the PMab-237 epitope is located in the PLD of wPDPN.

## Introduction

Podoplanin (PDPN)/T1alpha/Aggrus/PA2.26 is a type I transmembrane sialoglycoprotein consisting of a heavily glycosylated extracellular domain, a single transmembrane, and a short nine amino acid (AA) cytoplasmic tail.^(1–4)^ PDPN/Aggrus possesses the EDxxVTPG sequence at its N-terminus, which is known to be the platelet aggregation-stimulating (PLAG) domains (PLAG1, PLAG2, and PLAG3).^(3,5)^ In addition, the PLAG-like domain (PLD) of the E(D/E)xx(T/S)xx sequence, also known as PLAG4, has been reported to be present in the middle of PDPN.^(6,7)^ PLAG domains are highly conserved among mammalian PDPNs.^(7)^

PDPN is expressed in lymphatic endothelial cells and is not expressed in vascular endothelial cells.^(8)^ The interaction between PDPN^(3)^ on lymphatic endothelial cells and C-type lectin-like receptor-2 on platelets was shown to facilitate embryonic blood/lymphatic vessel separation.^(9)^ Because PDPN/T1alpha is expressed in type I alveolar cells but not in type II alveolar cells, it is used as a specific marker of type I alveolar cells.^(10)^ In recent studies, other functions of PDPN were reported. The PDPN-positive cells, with the immune cells after myocardial infarction, positively affect immune cell recruitment.^(11)^ The PDPN-positive stromal cells play a critical role in a network of immunofibroblasts, which can support the earliest phases of tertiary lymphoid structure establishment.^(12)^ The expression of PDPN in chorionic villous stromal cells is in two important placental pathologies: preeclampsia and hydatidiform mole.^(13)^ Moreover, PDPN is upregulated in many cancers and is involved in cancer metastasis and malignant progression.^(14–17)^ Recent reports showed that PDPN is related to a progression in oral epithelial dysplasia and oral squamous cell carcinoma through a co-expression with sex-determining region Y-related Homeo box gene 2.^(18,19)^ Therefore, PDPN possesses many pathophysiological functions in malignant tissues.

Recently, we developed a novel anti-whale PDPN (wPDPN) monoclonal antibody (mAb), PMab-237, using the Cell-Based Immunization and Screening (CBIS) method.^(20)^ The CBIS method was established in our previous study^(21)^ to produce mAbs using cell lines for immunization and screening. PMab-237 specifically detected wPDPN by flow cytometry, western blotting, and immunohistochemical analyses. PMab-237 also strongly stained pulmonary type I alveolar cells, renal podocytes, and lymphatic endothelial cells of the harbor porpoise by the immunohistochemical analysis.^(22)^ However, the binding epitope of PMab-237 for wPDPN remains unknown. This study aimed to identify the epitope of PMab-237 through flow cytometry using the deletion mutants and point mutants of wPDPN.

## Materials and Methods

### Production of wPDPN mutants

Synthesized DNA encoding wPDPN was subcloned into the pCAG vector (FUJIFILM Wako Pure Chemical Corporation, Osaka, Japan), and an N-terminal RIEDL tag was added.^(22)^ The RIEDL tag was derived from the five AA sequences (Arg-Ile-Glu-Asp-Leu) of human PDPN, which was detected by clone LpMab-7.^(23)^ Deletion mutants of the wPDPN sequence were produced using a HotStar HiFidelity Polymerase Kit (Qiagen, Inc., Hilden, Germany) with oligonucleotides. Substitutions of AAs to alanine in the wPDPN sequence were conducted by QuikChange Lightning Site-Directed Mutagenesis Kits (Agilent Technologies, Inc., Santa Clara, CA). PCR fragments bearing the desired mutations were inserted into the pCAG vector using an In-Fusion HD Cloning Kit (Takara Bio, Inc., Shiga, Japan).

### Cell lines and culture condition

Chinese hamster ovary (CHO)-K1 was obtained from the American Type Culture Collection (Manassas, VA). The wPDPN mutation plasmids containing the RIEDL tag were transfected into CHO-K1 cells using Lipofectamine LTX (Thermo Fisher Scientific, Inc., Waltham, MA). Transiently transfected cells with deletion mutants or point mutants were cultured in RPMI 1640 medium (Nacalai Tesque, Inc., Kyoto, Japan), supplemented with 10% heat-inactivated fetal bovine serum (FBS) (Thermo Fisher Scientific, Inc.), 100 U/mL of penicillin, 100 μg/mL of streptomycin, and 25 μg/mL of amphotericin B (Nacalai Tesque, Inc.) at 37°C in a humidified atmosphere of 5% CO_2_ and 95% air.

### Flow cytometry

Transiently transfected CHO-K1 cells were detached by 0.25% trypsin/1 mM of ethylenediaminetetraacetic acid (Nacalai Tesque, Inc.), and were collected using 10% FBS in RPMI 1640 medium. After washing with 0.1% bovine serum albumin and phosphate-buffered saline, the cells were incubated with an anti-wPDPN antibody (PMab-237^(22)^; 1 μg/mL) or an anti-RIEDL tag antibody (LpMab-7^(23)^; 1 μg/mL) for 30 minutes at 4°C. Alexa Fluor 488-conjugated anti-mouse IgG (1:1000; Cell Signaling Technology, Inc., Danvers, MA) for detection of PMab-237 and LpMab-7 were added to each cell and incubated for 30 minutes at 4°C. Fluorescence data were collected and analyzed using a Cell Analyzer EC800 (Sony Corp., Tokyo, Japan).

## Results

### Epitope analysis using deletion mutants of wPDPN

**FIG. 1.**
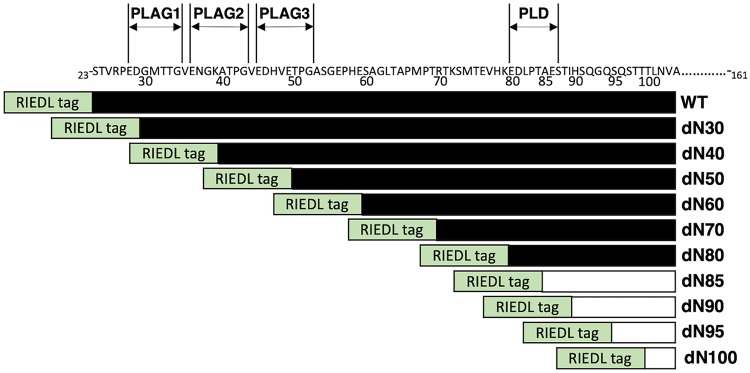
Schematic illustration of deletion mutants of anti-wPDPN. Illustration of WT and 10 deletion mutants of wPDPN: dN30, dN40, dN50, dN60, dN70, dN80, dN85, dN90, dN95, and dN100. Black bars: the positive reaction of PMab-237. White bars: the negative reaction of PMab-237. PLAG, platelet aggregation-stimulating; PLD, PLAG-like domain; wPDPN, whale podoplanin; WT, wild type.

Ten deletion mutants of wPDPN, such as dN30 (corresponding to 30–161 AA), dN40 (corresponding to 40–161 AA), dN50 (50–161 AA), dN60 (60–161 AA), dN70 (70–161 AA), dN80 (80–161 AA), dN85 (85–161 AA), dN90 (90–161 AA), dN95 (95–161 AA), and dN100 (100–161 AA), or wild type (WT) of wPDPN (corresponding to 23–161 AA) were generated using CHO-K1 cells (Fig. 1).

**FIG. 2.**
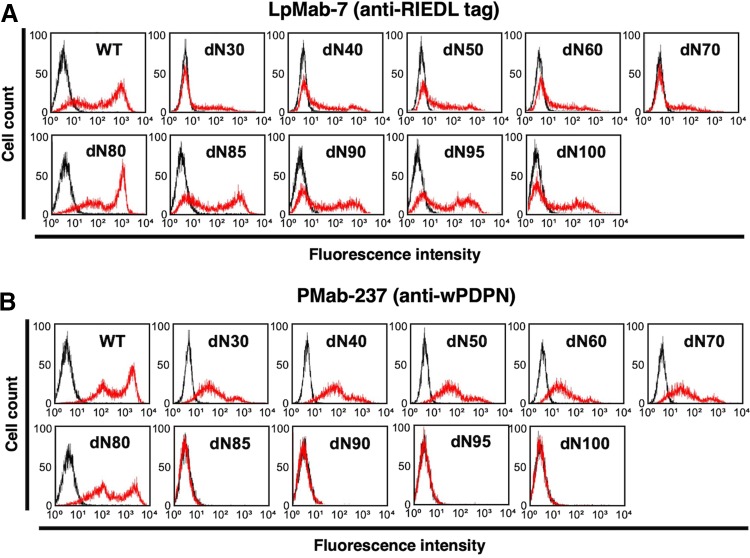
Epitope mapping of PMab-237 using deletion mutants of wPDPN. Deletion mutants of wPDPN were analyzed using flow cytometry. Deletion mutants were incubated with LpMab-7 **(**anti-RIEDL tag; red line, **A)**, PMab-237 (anti-wPDPN mAb; red line, **B**), or buffer control (black line, **A**, **B**) for 30 minutes at 4°C, followed by secondary antibodies. mAb, monoclonal antibody.

All deletion mutants and WT of wPDPN containing an N-terminal RIEDL tag were detected by LpMab-7 (an anti-RIEDL tag mAb), indicating that the expression level of each construct was high (Fig. 2A). In contrast, PMab-237 did not react with dN85, dN90, dN95, or dN100 (Fig. 2B), suggesting that the N-terminus of the PMab-237 epitope might exist between the 80th AA and 85th AA of wPDPN.

### Epitope analysis using point mutants of wPDPN

To identify the binding epitope of PMab-237, we then produced a series of point mutants of wPDPN using CHO-K1 cells, including E80A, D81A, L82A, P83A, T84A, A85G, E86A, S87A, T88A, I89A, H90A, S91A, Q92A, G93A, Q94A, S95A, Q96A, S97A, T98A, T99A, T100A, and L101A.

**FIG. 3.**
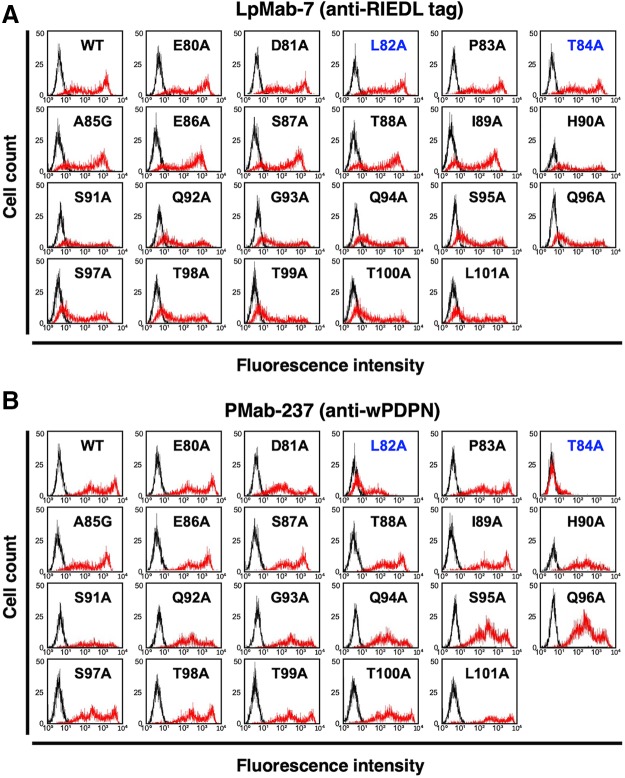
Epitope mapping of PMab-237 using point mutants of wPDPN. Transient point mutants expressing E80A, D81A, L82A, P83A, T84A, A85G, E86A, S87A, T88A, I89A, H90A, S91A, Q92A, G93A, Q94A, S95A, Q96A, S97A, T98A, T99A, T100A, and L101A of wPDPN were incubated with LpMab-7 **(**red line, **A)**, PMab-237 (red line, **B**), or buffer control (black line, **A**, **B**) for 30 minutes at 4°C, followed by secondary antibodies.

**FIG. 4.**
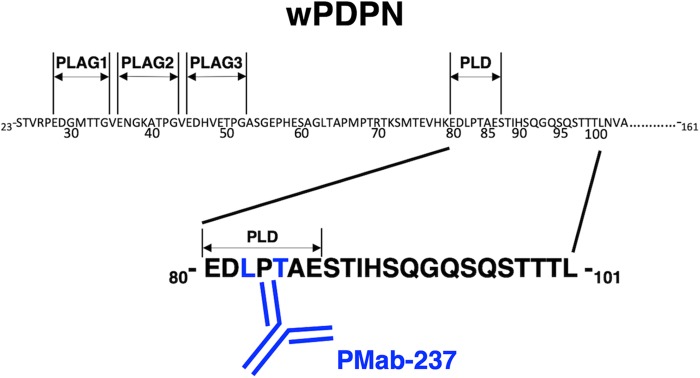
Schematic illustration of the epitope recognized by PMab-237. The critical epitope of PMab-237 includes Leu82 and Thr84 of wPDPN. Both Leu82 and Thr84 are included in the PLD.

LpMab-7 reacted with all point mutants, indicating that all transfectants express wPDPN (Fig. 3A). In contrast, PMab-237 weakly recognized L82A and did not react with T84A, indicating that the L82A and T84A of wPDPN could be included in the critical epitope of PMab-237 (Fig. 3B). Taken together, the epitope of PMab-237 is located in the PLD of wPDPN (Fig. 4).

## Discussion

We have demonstrated that the critical epitope of PMab-237 could include Leu82 and Thr84 of wPDPN using the deletion mutants and point mutants of wPDPN in CHO-K1 cells by flow cytometry. In our previous studies, we developed many mAbs against PDPNs of human,^(24)^ mouse,^(25)^ rat,^(26)^ rabbit,^(27)^ dog,^(28)^ cat,^(29)^ bovine,^(30)^ horse,^(31)^ Tasmanian devil,^(32)^ alpaca,^(33)^ bear,^(34)^ tiger,^(35)^ goat,^(36)^ pig,^(37,38)^ and whale.^(20)^ We successfully determined the binding epitope of those mAbs.^(22,34,39–48)^ These epitope mapping results showed that almost all anti-PDPN mAbs react with PLAG domains or PLDs.^(7,39–41,43,44,49–51)^ The critical epitope of PMab-237 was also shown to be located in PLD (Fig. 4), suggesting that PLAG domains and PLD were advantageous to the epitope for several applications such as flow cytometry, western blotting, and immunohistochemical analyses.

Furthermore, using glycan-deficient CHO cell lines, such as Lec1 (*N*-glycan-deficient), Lec2 (sialic acid-deficient), or Lec8 (galactose-deficient),^(52)^ we investigated whether the epitope of PMab-237 could include not only AAs but also glycans. A flow cytometric analysis demonstrated that PMab-237 reacts not only with CHO-K1/wPDPN but also with Lec1/wPDPN, Lec2/wPDPN, and Lec8/wPDPN (data not shown), indicating that glycans of wPDPN might not be included in the PMab-237 epitope. This analysis further suggests that no glycan is attached to Thr84 in the PLD of wPDPN (Fig. 4).

In conclusion, by using deletion mutants and point mutants of wPDPN in CHO-K1 cells, we have demonstrated that the critical epitope of PMab-237 may include Leu82 and Thr84 of wPDPN. PMab-237 could be a useful tool in elucidating the pathophysiological function of wPDPN.

## References

[1] Breiteneder-Geleff S, Matsui K, Soleiman A, Meraner P, Poczewski H, Kalt R, Schaffner G, and Kerjaschki D: Podoplanin, novel 43-kd membrane protein of glomerular epithelial cells, is down-regulated in puromycin nephrosis. Am J Pathol 1997;151:1141–1152 9327748 PMC1858024

[2] Ramirez MI, Rishi AK, Cao YX, and Williams MC: TGT3, thyroid transcription factor I, and Sp1 elements regulate transcriptional activity of the 1.3-kilobase pair promoter of T1alpha, a lung alveolar type I cell gene. J Biol Chem 1997;272:26285–26294 9334198 10.1074/jbc.272.42.26285

[3] Kato Y, Fujita N, Kunita A, Sato S, Kaneko M, Osawa M, and Tsuruo T: Molecular identification of Aggrus/T1alpha as a platelet aggregation-inducing factor expressed in colorectal tumors. J Biol Chem 2003;278:51599–51605 14522983 10.1074/jbc.M309935200

[4] Martin-Villar E, Scholl FG, Gamallo C, Yurrita MM, Munoz-Guerra M, Cruces J, and Quintanilla M: Characterization of human PA2.26 antigen (T1alpha-2, podoplanin), a small membrane mucin induced in oral squamous cell carcinomas. Int J Cancer 2005;113:899–910 15515019 10.1002/ijc.20656

[5] Kaneko MK, Kato Y, Kitano T, and Osawa M: Conservation of a platelet activating domain of Aggrus/podoplanin as a platelet aggregation-inducing factor. Gene 2006;378:52–57 16766141 10.1016/j.gene.2006.04.023

[6] Sayama Y, Sano M, Furusawa Y, Kaneko MK, and Kato Y: Epitope mapping of PMab-225 an anti-alpaca podoplanin monoclonal antibody using flow cytometry. Monoclon Antib Immunodiagn Immunother 2019;38:255–260 31613697 10.1089/mab.2019.0033

[7] Sekiguchi T, Takemoto A, Takagi S, Takatori K, Sato S, Takami M, and Fujita N: Targeting a novel domain in podoplanin for inhibiting platelet-mediated tumor metastasis. Oncotarget 2015;7:3934–3946 10.18632/oncotarget.6598 PMC4826181 26684030

[8] Breiteneder-Geleff S, Soleiman A, Kowalski H, Horvat R, Amann G, Kriehuber E, Diem K, Weninger W, Tschachler E, Alitalo K, and Kerjaschki D: Angiosarcomas express mixed endothelial phenotypes of blood and lymphatic capillaries: Podoplanin as a specific marker for lymphatic endothelium. Am J Pathol 1999;154:385–394 10027397 10.1016/S0002-9440(10)65285-6 PMC1849992

[9] Bertozzi CC, Schmaier AA, Mericko P, Hess PR, Zou Z, Chen M, Chen CY, Xu B, Lu MM, Zhou D, Sebzda E, Santore MT, Merianos DJ, Stadtfeld M, Flake AW, Graf T, Skoda R, Maltzman JS, Koretzky GA, and Kahn ML: Platelets regulate lymphatic vascular development through CLEC-2-SLP-76 signaling. Blood 2010;116:661–670 20363774 10.1182/blood-2010-02-270876 PMC3324297

[10] Fujino N, Kubo H, Ota C, Suzuki T, Suzuki S, Yamada M, Takahashi T, He M, Suzuki T, Kondo T, and Yamaya M: A novel method for isolating individual cellular components from the adult human distal lung. Am J Respir Cell Mol Biol 2012;46:422–430 22033268 10.1165/rcmb.2011-0172OC

[11] Cimini M, Garikipati VNS, de Lucia C, Cheng Z, Wang C, Truongcao MM, Lucchese AM, Roy R, Benedict C, Goukassian DA, Koch WJ, and Kishore R: Podoplanin neutralization improves cardiac remodeling and function after acute myocardial infarction. JCI Insight 2019;5:126967 31287805 10.1172/jci.insight.126967 PMC6693826

[12] Nayar S, Campos J, Smith CG, Iannizzotto V, Gardner DH, Mourcin F, Roulois D, Turner J, Sylvestre M, Asam S, Glaysher B, Bowman SJ, Fearon DT, Filer A, Tarte K, Luther SA, Fisher BA, Buckley CD, Coles MC, and Barone F: Immunofibroblasts are pivotal drivers of tertiary lymphoid structure formation and local pathology. Proc Natl Acad Sci U S A 2019;116:13490–13497 31213547 10.1073/pnas.1905301116 PMC6613169

[13] Onak Kandemir N, Barut F, Barut A, Birol IE, Dogan Gun B, and Ozdamar SO: Biological importance of podoplanin expression in chorionic villous stromal cells and its relationship to placental pathologies. Sci Rep 2019;9:14230 31578434 10.1038/s41598-019-50652-9 PMC6775148

[14] Quintanilla M, Montero-Montero L, Renart J, and Martin-Villar E: Podoplanin in inflammation and cancer. Int J Mol Sci 2019;20:E707 30736372 10.3390/ijms20030707 PMC6386838

[15] Kunita A, Kashima TG, Morishita Y, Fukayama M, Kato Y, Tsuruo T, and Fujita N: The platelet aggregation-inducing factor aggrus/podoplanin promotes pulmonary metastasis. Am J Pathol 2007;170:1337–1347 17392172 10.2353/ajpath.2007.060790 PMC1829466

[16] Kimura N, and Kimura I: Podoplanin as a marker for mesothelioma. Pathol Int 2005;55:83–86 15693854 10.1111/j.1440-1827.2005.01791.x

[17] Kato Y, Kaneko M, Sata M, Fujita N, Tsuruo T, and Osawa M: Enhanced expression of Aggrus (T1alpha/podoplanin), a platelet-aggregation-inducing factor in lung squamous cell carcinoma. Tumor Biol 2005;26:195–200 10.1159/000086952 16006773

[18] Verma V, and Chandrashekar C: Evaluation of SOX2 and podoplanin expression in oral epithelial dysplasia and its correlation with malignant transformation. J Investig Clin Dent 2019:10:e12450 10.1111/jicd.12450 31464104

[19] Pradhan S, Guddattu V, and Solomon MC: Association of the co-expression of SOX2 and podoplanin in the progression of oral squamous cell carcinomas—An immunohistochemical study. J Appl Oral Sci 2019;27:e20180348 31508790 10.1590/1678-7757-2018-0348 PMC9648958

[20] Kato Y, Furusawa Y, Itai S, Takei J, Nakamura T, Sano M, Harada H, Yamada S, and Kaneko MK: Establishment of an anticetacean podoplanin monoclonal antibody PMab-237 for immunohistochemical analysis. Monoclon Antib Immunodiagn Immunother 2019;38:108–113 31161965 10.1089/mab.2019.0013

[21] Yamada S, Itai S, Nakamura T, Yanaka M, Kaneko MK, and Kato Y: Detection of high CD44 expression in oral cancers using the novel monoclonal antibody, C44Mab-5. Biochem Biophys Rep 2018;14:64–68 29872736 10.1016/j.bbrep.2018.03.007 PMC5986985

[22] Yamada S, Itai S, Nakamura T, Takei J, Sano M, Konnai S, Kobayashi A, Nakagun S, Kobayashi Y, Kaneko MK, and Kato Y: Immunohistochemical analysis of the harbor porpoise using antipodoplanin antibody PMab-237. Monoclon Antib Immunodiagn Immunother 2019;38:104–107 31161964 10.1089/mab.2019.0014

[23] Oki H, Kaneko MK, Ogasawara S, Tsujimoto Y, Liu X, Sugawara M, Takakubo Y, Takagi M, and Kato Y: Characterization of a monoclonal antibody LpMab-7 recognizing non-PLAG domain of podoplanin. Monoclon Antib Immunodiagn Immunother 2015;34:174–180 26090595 10.1089/mab.2014.0090

[24] Kato Y, Kaneko MK, Kuno A, Uchiyama N, Amano K, Chiba Y, Hasegawa Y, Hirabayashi J, Narimatsu H, Mishima K, and Osawa M: Inhibition of tumor cell-induced platelet aggregation using a novel anti-podoplanin antibody reacting with its platelet-aggregation-stimulating domain. Biochem Biophys Res Commun 2006;349:1301–1307 16979138 10.1016/j.bbrc.2006.08.171

[25] Kaji C, Tsujimoto Y, Kato Kaneko M, Kato Y, and Sawa Y: Immunohistochemical examination of novel rat monoclonal antibodies against mouse and human podoplanin. Acta Histochem Cytochem 2012;45:227–237 23012488 10.1267/ahc.12008 PMC3445762

[26] Oki H, Honma R, Ogasawara S, Fujii Y, Liu X, Takagi M, Kaneko MK, and Kato Y: Development of sensitive monoclonal antibody PMab-2 against rat podoplanin. Monoclon Antib Immunodiagn Immunother 2015;34:396–403 26683179 10.1089/mab.2015.0041

[27] Honma R, Fujii Y, Ogasawara S, Oki H, Liu X, Nakamura T, Kaneko MK, Takagi M, and Kato Y: Establishment of a novel monoclonal antibody PMab-32 against rabbit podoplanin. Monoclon Antib Immunodiagn Immunother 2016;35:41–47 26788987 10.1089/mab.2015.0066

[28] Honma R, Kaneko MK, Ogasawara S, Fujii Y, Konnai S, Takagi M, and Kato Y: Specific detection of dog podoplanin expressed in renal glomerulus by a novel monoclonal antibody PMab-38 in immunohistochemistry. Monoclon Antib Immunodiagn Immunother 2016;35:212–216 27380308 10.1089/mab.2016.0022

[29] Yamada S, Itai S, Nakamura T, Yanaka M, Saidoh N, Chang YW, Handa S, Harada H, Kagawa Y, Ichii O, Konnai S, Kaneko MK, and Kato Y: PMab-52: Specific and sensitive monoclonal antibody against cat podoplanin for immunohistochemistry. Monoclon Antib Immunodiagn Immunother 2017;36:224–230 28737447 10.1089/mab.2017.0027

[30] Honma R, Ogasawara S, Kaneko M, Fujii Y, Oki H, Nakamura T, Takagi M, Konnai S, and Kato Y: PMab-44 detects bovine podoplanin in immunohistochemistry. Monoclon Antib Immunodiagn Immunother 2016;35:186–190 27267652 10.1089/mab.2016.0016

[31] Furusawa Y, Yamada S, Itai S, Nakamura T, Yanaka M, Sano M, Harada H, Fukui M, Kaneko MK, and Kato Y: PMab-219: A monoclonal antibody for the immunohistochemical analysis of horse podoplanin. Biochem Biophys Rep 2019;18:100616 30766925 10.1016/j.bbrep.2019.01.009 PMC6360987

[32] Furusawa Y, Yamada S, Itai S, Nakamura T, Takei J, Sano M, Harada H, Fukui M, Kaneko MK, and Kato Y: Establishment of a monoclonal antibody PMab-233 for immunohistochemical analysis against Tasmanian devil podoplanin. Biochem Biophys Rep 2019;18:100631 30984883 10.1016/j.bbrep.2019.100631 PMC6446048

[33] Kato Y, Furusawa Y, Yamada S, Itai S, Takei J, Sano M, and Kaneko MK: Establishment of a monoclonal antibody PMab-225 against alpaca podoplanin for immunohistochemical analyses. Biochem Biophys Rep 2019;18:100633 30997422 10.1016/j.bbrep.2019.100633 PMC6451175

[34] Furusawa Y, Takei J, Sayama Y, Yamada S, Kaneko MK, and Kato Y: Development of an anti-bear podoplanin monoclonal antibody PMab-247 for immunohistochemical analysis. Biochem Biophys Rep 2019;18:100644 31061899 10.1016/j.bbrep.2019.100644 PMC6488525

[35] Furusawa Y, Kaneko MK, Nakamura T, Itai S, Fukui M, Harada H, Yamada S, and Kato Y: Establishment of a monoclonal antibody PMab-231 for tiger podoplanin. Monoclon Antib Immunodiagn Immunother 2019;38:89–95 31009336 10.1089/mab.2019.0003

[36] Furusawa Y, Yamada S, Nakamura T, Sano M, Sayama Y, Itai S, Takei J, Harada H, Fukui M, Kaneko MK, and Kato Y: PMab-235: A monoclonal antibody for immunohistochemical analysis against goat podoplanin. Heliyon 2019;5:e02063 31338471 10.1016/j.heliyon.2019.e02063 PMC6626078

[37] Kato Y, Yamada S, Furusawa Y, Itai S, Nakamura T, Yanaka M, Sano M, Harada H, Fukui M, and Kaneko MK: PMab-213: A monoclonal antibody for immunohistochemical analysis against pig podoplanin. Monoclon Antib Immunodiagn Immunother 2019;38:18–24 30802179 10.1089/mab.2018.0048

[38] Furusawa Y, Yamada S, Itai S, Sano M, Nakamura T, Yanaka M, Fukui M, Harada H, Mizuno T, Sakai Y, Takasu M, Kaneko MK, and Kato Y: PMab-210: A monoclonal antibody against pig podoplanin. Monoclon Antib Immunodiagn Immunother 2019;38:30–36 30681406 10.1089/mab.2018.0038

[39] Yamada S, Itai S, Furusawa Y, Kaneko MK, and Kato Y: Epitope mapping of anti-pig podoplanin monoclonal antibody PMab-213. Monoclon Antib Immunodiagn Immunother 2019;38:224–229 31464569 10.1089/mab.2019.0023

[40] Takei J, Itai S, Furusawa Y, Yamada S, Nakamura T, Sano M, Harada H, Fukui M, Kaneko MK, and Kato Y: Epitope mapping of anti-tiger podoplanin monoclonal antibody PMab-231. Monoclon Antib Immunodiagn Immunother 2019;38:129–132 31112076 10.1089/mab.2019.0012

[41] Kaneko MK, Furusawa Y, Sano M, Itai S, Takei J, Harada H, Fukui M, Yamada S, and Kato Y: Epitope mapping of the antihorse podoplanin monoclonal antibody PMab-202. Monoclon Antib Immunodiagn Immunother 2019;38:79–84 30939066 10.1089/mab.2019.0001

[42] Yamada S, Kaneko MK, Itai S, Chang YW, Nakamura T, Yanaka M, Ogasawara S, Murata T, Uchida H, Tahara H, Harada H, and Kato Y: Epitope mapping of monoclonal antibody PMab-48 against dog podoplanin. Monoclon Antib Immunodiagn Immunother 2018;37:162–165 29608407 10.1089/mab.2018.0006

[43] Yamada S, Itai S, Kaneko MK, Konnai S, and Kato Y: Epitope mapping of anti-mouse podoplanin monoclonal antibody PMab-1. Biochem Biophys Rep 2018;15:52–56 29998193 10.1016/j.bbrep.2018.07.002 PMC6039309

[44] Chang YW, Kaneko MK, Yamada S, and Kato Y: Epitope mapping of monoclonal antibody PMab-52 against cat podoplanin. Monoclon Antib Immunodiagn Immunother 2018;37:95–99 29394131 10.1089/mab.2017.0067

[45] Chang YW, Yamada S, Kaneko MK, and Kato Y: Epitope mapping of monoclonal antibody PMab-38 against dog podoplanin. Monoclon Antib Immunodiagn Immunother 2017;36:291–295 29161183 10.1089/mab.2017.0048

[46] Ogasawara S, Kaneko MK, Price JE, and Kato Y: Characterization of anti-podoplanin monoclonal antibodies: Critical epitopes for neutralizing the interaction between podoplanin and CLEC-2. Hybridoma 2008;27:259–267 18707544 10.1089/hyb.2008.0017

[47] Takei J, Furusawa Y, Yamada S, Nakamura T, Sayama Y, Sano M, Konnai S, Kobayashi A, Harada H, Kaneko MK, and Kato Y: PMab-247 detects bear podoplanin in immunohistochemical analysis. Monoclon Antib Immunodiagn Immunother 2019;38:171–174 31313968 10.1089/mab.2019.0019

[48] Sano M, Kaneko MK, and Kato Y: Epitope mapping of monoclonal antibody PMab-233 against Tasmanian devil podoplanin. Monoclon Antib Immunodiagn Immunother 2019;38:261–265 31621497 10.1089/mab.2019.0032

[49] Takei J, Itai S, Harada H, Furusawa Y, Miwa T, Fukui M, Nakamura T, Sano M, Sayama Y, Yanaka M, Handa S, Hisamatsu K, Nakamura Y, Yamada S, Kaneko MK, and Kato Y: Characterization of anti-goat podoplanin monoclonal antibody PMab-235 using immunohistochemistry against goat tissues. Monoclon Antib Immunodiagn Immunother 2019;38:213–219 31403389 10.1089/mab.2019.0022

[50] Furusawa Y, Yamada S, Itai S, Nakamura T, Fukui M, Harada H, Kaneko MK, and Kato Y: Elucidation of critical epitope of anti-rat podoplanin monoclonal antibody PMab-2. Monoclon Antib Immunodiagn Immunother 2018;37:188–193 30088964 10.1089/mab.2018.0025 PMC6121180

[51] Yamada S, Honma R, Kaneko MK, Nakamura T, Yanaka M, Saidoh N, Takagi M, Konnai S, and Kato Y: Characterization of the anti-bovine podoplanin monoclonal antibody PMab-44. Monoclon Antib Immunodiagn Immunother 2017;36:129–134 28498096 10.1089/mab.2017.0016

[52] Kaneko M, Kato Y, Kunita A, Fujita N, Tsuruo T, and Osawa M: Functional sialylated O-glycan to platelet aggregation on Aggrus (T1alpha/podoplanin) molecules expressed in Chinese hamster ovary cells. J Biol Chem 2004;279:38838–38843 15231832 10.1074/jbc.M407210200

